# Genome Sequences of Two Plant-Beneficial *Chryseobacterium* Strains Isolated from Agricultural Soils in the Province of Quebec, Canada

**DOI:** 10.1128/mra.00104-23

**Published:** 2023-06-15

**Authors:** Adrien Biessy, Marie Ciotola, Mélanie Cadieux, Daphné Albert, Martin Filion

**Affiliations:** a Saint-Jean-sur-Richelieu Research and Development Centre, Agriculture and Agri-Food Canada, Saint-Jean-sur-Richelieu, Quebec, Canada; University of Delaware College of Engineering

## Abstract

Two *Chryseobacterium* strains, B21-013 and B21-037, were isolated from agricultural soils located in the province of Quebec, Canada, as part of a screening for plant-beneficial bacteria able to suppress Xanthomonas hortorum pv. *vitians* and other lettuce bacterial pathogens. Here, we report the genome sequences of these two organisms.

## ANNOUNCEMENT

Members of the genus *Chryseobacterium* are rod-shaped, non-spore forming, nonmotile Gram-negative bacteria that are found in various environments (1, 2). Some species are associated with human illness (3, 4), while others are found in the rhizosphere and soil environments (5, 6). Several *Chryseobacterium* strains were previously shown to display plant growth promotion and biocontrol activity towards several plant pathogens, including Phytophthora capsici (7, 8).

Two *Chryseobacterium* strains (B21-013 and B21-037) were isolated from agricultural fields located in the Montérégie region (Quebec, Canada) as part of a search for plant-beneficial bacteria able to suppress various lettuce bacterial pathogens. These two strains were isolated as previously described (9). Briefly, 10 soil samples were collected as close as possible to the plant roots of various vegetable crop species grown on two agricultural farms located in Dunham and Brigham (Quebec, Canada). The soil samples were pooled, and 1 g of soil was added to 100 mL of a 0.9% NaCl solution. The suspension was shaken, serially diluted, and plated onto King’s B agar (10) supplemented with cycloheximide (100 μg mL^−1^), ampicillin (40 μg mL^−1^), and chloramphenicol (13 μg mL^−1^). The plates were incubated at 25°C for 48 h, and the isolated colonies were subsequently purified on the same medium. The strains were cryopreserved at −80°C in tryptic soy broth (BD Biosciences) containing 10% glycerol (vol/vol). The two strains were grown in King’s B agar for 48 h at 25°C, and genomic DNA was extracted using the DNeasy UltraClean microbial kit (Qiagen) according to the manufacturer’s instructions. Library preparation and genome sequencing were performed at the Integrated Microbiome Resource (Halifax, Nova Scotia, Canada). Genomic DNA was mechanically sheared into DNA fragments with a target size of 10 kb using g-TUBE devices (Covaris). The libraries were constructed using the PacBio SMRTbell Express template prep kit (Pacific Biosciences). Genome sequencing was carried out on a PacBio Sequel sequencer (v3 chemistry). The quality of the raw reads was checked using FastQC v0.11.9 (11), and genome assembly was performed using Flye v2.8.1 (12) in “–pacbio-raw” mode. The high-quality draft genomes of B21-013 and B21-037 comprised 2 and 3 contigs, respectively. The two genomes were annotated using the NCBI Prokaryotic Genome Annotation Pipeline v6.0 (13). Default parameters were used for all software. Read and assembly metrics, as well as general genome features, are presented in Table 1.

**TABLE 1:** Sequencing, assembly, and annotation metrics

Metric	Data for strain:	
	B21-013	B21-037
No. of reads	317,044	1,144,918
Read *N*_50_ (bp)	8,809	4,251
Coverage (×)	304	774
Genome size (Mb)	5.40	4.95
GC content (%)	36.2	36.1
No. of contigs	2	3
Contig *N*_50_ (Mb)	5.39	4.92
No. of CDSs^*a*^	4,694	4,351
No. of pseudogenes	46	69
No. of rRNAs	21	18
No. of tRNAs	90	70
GenBank accession no.	JALGCC000000000	JALGCB000000000
SRA accession no.	SRR23348883	SRR23348882

a CDSs, coding DNA sequences.

**FIG 1:**
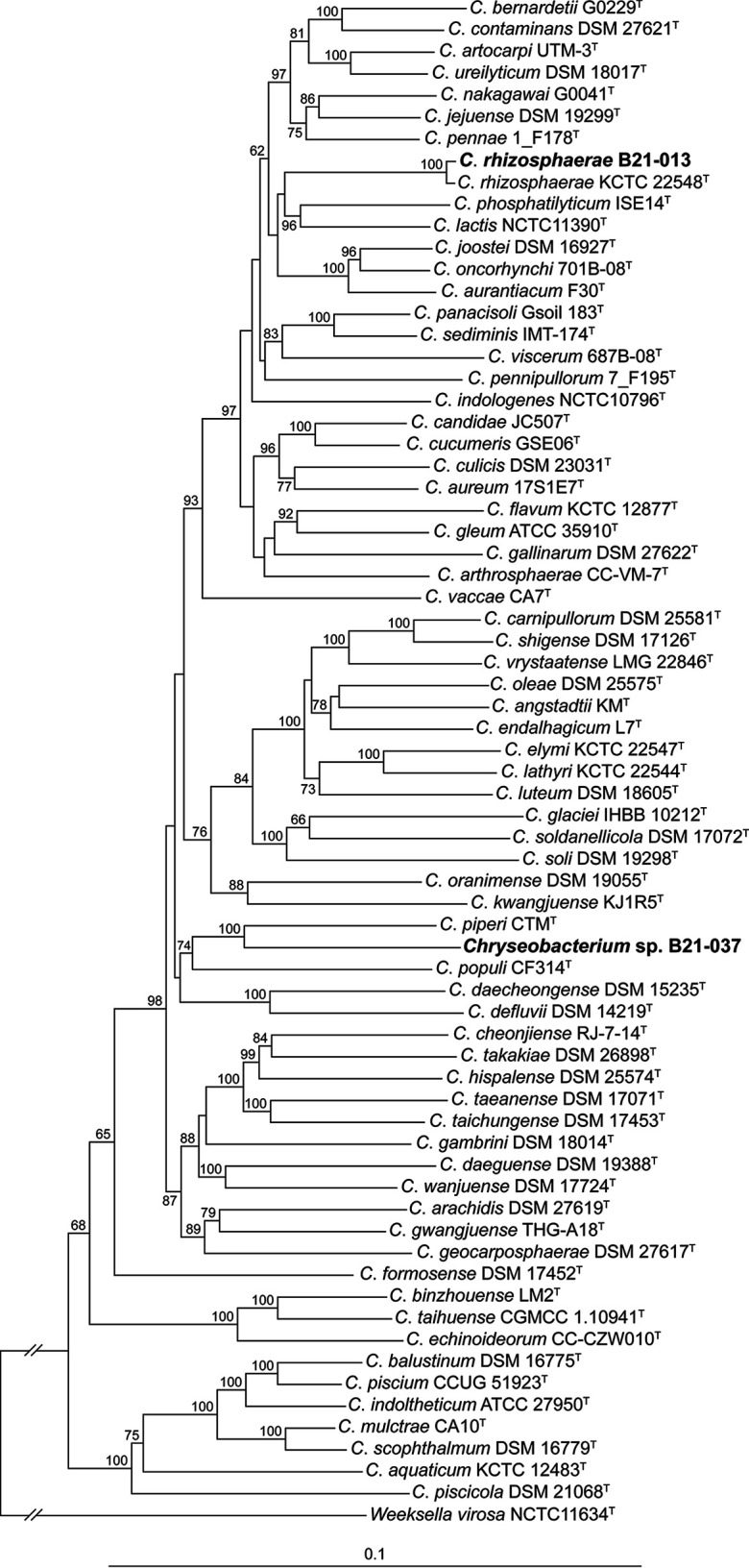
Neighbor-joining phylogeny of the genus *Chryseobacterium*. The complete nucleotide sequences of four housekeeping genes (16S rRNA gene, *gyrB*, *rpoD*, and *rpoB*) were concatenated and subsequently aligned using MUSCLE v3.8.425 (16). The phylogenetic tree was generated from the entire alignment using the Geneious tree builder (Biomatters) with the Jukes-Cantor method. The two strains whose genomes are reported in this announcement are highlighted in bold. Only bootstrap values above 60% (from 1,000 replicates) are shown. Weeksella virosa NCTC11634^T^ was used as an outgroup.

To gain insight into the phylogenetic relationships between these two strains and other previously described *Chryseobacterium* species, a multilocus sequence analysis with four housekeeping genes (16S rRNA gene, *gyrB*, *rpoD*, and *rpoB*) was performed (Fig. 1). B21-013 was shown to be closely related to Chryseobacterium rhizosphaerae KCTC 22548^T^. B21-037 was found to cluster with Chryseobacterium piperi CTM^T^. Species-level identification was performed using the Type (Strain) Genome Server (14, 15). Digital DNA-DNA hybridization (dDDH) values between the two strains under study and various *Chryseobacterium* type strains were calculated using the d_4_ formula (on 8 February 2023). B21-013 was found to belong to the species *C*. *rhizosphaerae* (dDDH = 86.6% with KCTC 22548^T^). B21-037 does not belong to the species *C*. *piperi* (dDDH = 23.8% with CTM^T^) or to any other validly named species.

### Data availability.

The complete genomes of strains B21-013 and B21-037 have been deposited at DDBJ/ENA/GenBank under the following accession numbers: JALGCC000000000 (B21-013) and JALGCB000000000 (B21-037). The raw sequencing reads have been deposited in the Sequence Read Archive (BioProject accession number PRJNA814203) under the following accession numbers: SRR23348883 (B21-013) and SRR23348882 (B21-037). The versions described in this paper are the first versions.
